# Mental health of unaccompanied children: effects of U.S. immigration policies

**DOI:** 10.1192/bjo.2021.1016

**Published:** 2021-11-02

**Authors:** Suzan J. Song

**Affiliations:** Department of Psychiatry and Behavioral Sciences, George Washington University, Washington, DC, USA

**Keywords:** Refugee, asylum, mental health, children, migrant

## Abstract

**Background:**

There is an unprecedented surge of forcibly displaced people globally, with a crisis of unaccompanied minors seeking haven across the US border.

**Aims:**

This paper aims to provide an understanding of the intersection between mental health and immigration policies.

**Method:**

Examples of contemporary policies that focus on the deterrence, detention and deportation of unaccompanied minors in the USA, will be discussed, as well as the mental health effects of such ‘iron triangle’ immigration policies.

**Results:**

In the ideal circumstances, systems and policies for migrant children would uphold international humanitarian law, hasten the shift from enforcement to protection, adhere to a ‘do no (further) harm’ model that uses a trauma-informed, culturally responsive approach to engaging with migrant children, engage the community as stakeholders to end detention and advocate to share the burden of responsibility.

**Conclusions:**

Building a humanitarian response that protects both country and migrant interest is possible through commitment and policy change that addresses mental, physical and legal protection needs.

Around the world, the forced displacement of children because of economic and political instability, disasters, war and armed conflict, and chronic violence have surged. Approximately half of the world's refugees are younger than 18 years old and an estimated 33 million children are forcibly displaced from their homes, 1.5 million of whom are seeking asylum.^1^ Although the 1951 Convention on Refugees by the United Nations seals the obligation to protect those fleeing war and persecution into international law, rising xenophobia and management of emergent COVID-19 public health measures have made the humanitarian needs of displaced children and families highly political and controversial.

Asylum seekers are defined as people who seek protection under the Convention on Refugees after entering another country on a temporary visa or without other documents. Often, unaccompanied minors, defined as children under 18 years old who arrive without a legal guardian, leave their homes seeking asylum in a new country. Governments around the world have long been divided on the question of how to respond to displaced people seeking safe haven and legal protections. The debate over asylum procedures for unaccompanied children in particular, has become a highly controversial issue with outspoken government officials, politicians, lawmakers and advocates in gridlock, but with little mention of the mental health and well-being of these children.

The focus of this article is to provide an overview of recent immigration policies and the mental health of unaccompanied children seeking asylum. I will give examples of US immigration policies and procedures as well as discuss the mental health and well-being of unaccompanied children, to encourage governments to respect international law and multilateral treaties. The US government has historically respected the Convention on Refugees that provides that any person who arrives in the USA may apply for asylum. However, over recent years, policies have eroded the US immigration system and caused further harm to those seeking protection.

## Method

This is a descriptive study. Examples of contemporary policies that focus on the deterrence, detention and deportation of unaccompanied minors in the USA, are discussed, as well as the mental health effects of such ‘iron triangle’ immigration policies.

## Results

### Burden of proof and mental health for unaccompanied children

The composition of people crossing the US border seeking asylum has changed over the past decade from mostly single men from Mexico seeking employment opportunities to children and families accounting now for more than half of all people crossing the border, with numbers quadrupling between 2019 and 2020.^2^ These stark changes in migration profiles reflect worsening sociopolitical landscapes and associated violence within the neighbouring countries of El Salvador, Guatemala and Honduras (the northern triangle of Central America). In recent years, the majority of unaccompanied children entering the USA are 13- to 17-year-olds^3^ and may be eligible for other forms of protection such as T and U visas, and special immigrant juvenile status.

According to the Convention on Refugees, in order to justify a refugee claim, an applicant must prove that ‘owing to a well-founded fear of being persecuted for reasons of race, religion, nationality, membership of a particular group or political opinion, (he/she) is outside the country of his nationality and is unable to or unwilling to avail himself of the protection of that country …’^4^ Although children must meet this same standard of evidence of fear of persecution that adults do, children developmentally struggle to categorise their experiences into a legal group that makes them eligible for asylum. The legal language and cultural shaping of their experiences, in addition to the need to prove that their country's government was unwilling to protect them, puts an undue and developmentally inappropriate burden on a child. Moreover, although many children enter the US after forced coercion into gang involvement, retaliation for gang involvement is not explicitly identified in asylum guidelines, often rendering asylum unsuccessful.^5^

This burden of proof creates an added stress to the mental health of this population. Unaccompanied children who seek asylum are looking for safety and protections from life-threatening conditions that may be associated with trafficking, war, organised violence, and political or religious unrest that can expose children to potentially traumatic events such as extreme violence, torture, imprisonment and killings in addition to loss of family, safety and security. In addition to pre-migration factors such as exposure to human trafficking and torture, post-migration factors such as prolonged asylum procedures, poverty and poor housing are also associated with poor mental health.^6^ Studies on unaccompanied children have shown widely variable prevalence rates for post-traumatic stress disorder (17–85%), depression (13–76%) and anxiety (11%–85%), as well as behavioural and conduct, psychotic and somatic disorders.^7^ The variance in studies can be explained in part by the differences within samples, self-report versus clinical assessment methodologies, and lack of culturally validated measures with appropriate cut-offs. Despite the wide variance, the consensus is that psychological difficulties are higher in unaccompanied children than the general population.

### Controversy around asylum: enforcement over protection

As policies relating to asylum vary across the world, several key concerns have been raised about the management of unaccompanied children. Whereas immigration policies affect the mental health of children fleeing persecution, many policies are not developed with a child's mental health or well-being in mind.^8^ Asylum procedures are often guided by concerns about enforcement instead of protection. Tasked with a law enforcement mandate, Customs and Border Patrol (CBP) facilities have been described as ‘inhumane,’ with references to lack of bedding and bathing facilities, inadequate access to food and water, open toilets, confiscation of belongings and lack of access to essential medical care, sexual violence by staff against children in custody, inappropriate use of solitary confinement, and lack of timely medical treatment contributing to the death of at least nine children under immigration custody since 2018.^9^ The debate about enforcement versus protection has been inflamed with the thousands of unaccompanied children who have presented themselves to government officials at the border, creating a logistical, political and ethical challenge.

### Iron triangle of deterrence

In US politics, the ‘iron triangle’ refers to congressional committees, bureaucracy and interest groups influencing each other to create and consolidate power. In this article, the iron triangle of deterrence is used to describe how deterrence, deportation and detention are used as leverage points to enact power. Many policies overlap in these pillars, and implicit in the 2016–2020 iron triangle of policies is deterring migrants from crossing borders.

#### Deterrence: refugee admissions and asylum processing

In the context of children and families seeking asylum at the US–Mexico border, federal policies guiding detention practices responsively evolved with the goal of immigration deterrence. Starting in 2017, the US administration sharply curbed refugee admissions every year until 2019, when the number was capped at 15 000, the lowest since the 1980 Refugee Act took effect.^10^ As the numbers of refugees legally permitted entry into the US plummeted, the number of unaccompanied children attempting to cross the border increased as they tried to find alternative ways to safety. A surge of migrants at the southern US/Mexico border has led to an excess of one million pending asylum cases in US immigration courts.^11^

This backlog of cases is amplified because of a change in the asylum process that substitutes the first-in, first-out policy for the last-in, first-out policy. Since 2014, the US Citizen and Immigration Services scheduled asylum interviews in the order they were received – the longest pending applications scheduled first, allowing applicants to file the ‘bare bones’ application, then gather relevant evidence and supporting documents while awaiting months or years for the interview. In January 2018, asylum interviews were scheduled starting with the newest filings and working back towards older ones, leaving applicants little time to prepare their evidence.

Another bottleneck to the immigration process is the swift appointment of judges. Under the 2016 administration, immigration judges were allowed temporary appointments before background investigations were complete, leading the American Bar Association to report that the process may have allowed ‘underqualified or potentially biased judges to be hired due to lack of thorough vetting’.^12^ These temporary immigration judges were twice as likely to have military experience, linked to higher rate of deportation orders, and 42% had no immigration experience – double those of the preceding cohort of judges.^13^ Jack Weil, a senior justice department judge responsible for training the nation's immigration judges, stated in his sworn testimony, ‘I've taught immigration law literally to 3-year-olds and 4-year-olds. It takes a lot of time. It takes a lot of patience. They get it. It's not the most efficient, but it can be done’.^14^ His statement shows how misinformation about child development can shape policies that promote having children legally represent themselves in immigration court.

#### Detention: management of children and families

The United Nations Convention on the Rights of the Child, an internationally recognised framework for the protection of children's basic rights, emphasises freedom from arbitrary arrest and detention (Article 37), special protection to child asylum seekers (Article 22), humane and appropriate treatment of children in detention (Article 37), and guidelines on protecting family unity (Article 9).^15^ Although every country in the world has ratified the Convention on the Rights of the Child except the USA, the detention of children is condemned by professional organisations within the USA.^16^ Despite this, the USA has the largest system of immigration detention facilities, growing twenty-fold since 1979.^17^ When children come to the border, they are placed into CBP custody. Under the Trafficking Victims Protection Reauthorization Act, unaccompanied children must be transferred within 72 h from CBP custody to the Office of Refugee Resettlement that manages 170 shelters, group homes, foster care and therapeutic facilities across the country. In 2019, the Office of Inspector General reviewed five border patrol facilities and found overcrowding with 31% of children in CBP detention held for longer than 72 h, some younger than 7 years old.^18^

The increase of children and families in immigration detention was amplified by the ‘zero tolerance’ policy in April 2018, which criminalised crossing the border to seek asylum.^19^ The 1997 Reno *v*. Flores Settlement Agreement originally codified legal protections criteria for the detention and treatment of minors in immigration custody and made the detention of minors for more than 20 days illegal.^20^ In the setting of lengthy criminal prosecution procedures under ‘zero tolerance’, children (including infants and toddlers) were separated indefinitely from caretakers and placed into detention. Between July 2017 and June 2018, more than 5400 children were estimated to have been separated from their caregivers, the majority aged 12 years and younger.^21^ Shortly thereafter, a judgment arising from a class action lawsuit deemed the separation of families unconstitutional in denying due process and mandated the identification and reunification of families. Unfortunately, processes for tracing and reunification of forcibly separated children in the US lacked transparency, standardisation and resources, resulting in prolonged separations and poor follow-up of separated children.^22^ As of December 2020, parents of 628 children were still missing after 3 years, at least 60 of those children were under 5 years old at the time of separation.^23^

#### Deportation and expulsion: migrant protection policy and Title 42

Deportations markedly increased over the past few decades in the USA until the COVID-19 pandemic in 2020 that upended the country's immigration system. In March 2020, the administration cited a 1944 statute that gives the executive branch power to block foreigners from entering the country for public health protection. The Center for Disease Control closed the US border to asylum seekers over objections of their own medical experts.^24^ This order, ‘Title 42’ was used to justify the automatic summary expulsions for children and families at the border and already under US custody who did not have entry documents.^25^ Title 42 builds on the Migration Protection Protocol (MPP) policy from January 2019, which authorised all asylum seekers to be sent to Mexico to await hearings. These are not considered ‘deportation’ as migrants are not allowed the right to present their case before an immigration judge, and most are returned to Mexico within hours. Between March and November 2020, most unaccompanied children were turned away because of Title 42, with an estimated 15 800 children expelled to Mexico via the MPP to await asylum claims outside the US border.^26^

The new administration decided that unaccompanied children would not be subjected to Title 42 but continued the practice for adults. Quickly, the number of unaccompanied children in need of processing increased, overwhelming the US shelter capacity that was reduced during the past administration because of low numbers of migrant children processed and to allow for COVID-19-related public health measures. Presently, human rights advocates continue to challenge these policies, maintaining that the Title 42 order has no basis in public health science, and discriminates by focusing on asylum seekers while allowing hundreds of thousands of other border crossings to take place.^27^ There is no evidence that refugees are responsible for the spread of infectious disease and migrants do not represent a burden to host country healthcare systems.

### Mental health of unaccompanied children

There is a human cost to these contemporary immigration policies affecting unaccompanied children seeking asylum. The prolonged uncertainty that accompanies awaiting determination of asylum status has profound effects in creating a sense of loss of control and powerlessness, and is associated with poor mental health.^28^ Children awaiting asylum are forced to put their lives on hold as they are challenged in considering future options. The liminal space of belonging for children who have left behind families, supports and identities, but have yet to arrive in the safety of stable housing, legal status or protection, can exacerbate their pre-existing vulnerability to mental health problems because of higher exposures to traumatic effects before and during migration. This adds to the ‘building block effect’ where uncertainty and insecurity add to a cumulative effect of exposure to trauma that is associated with an increase in mental health problems such as post-traumatic stress disorder.^29^

Moreover, multiple studies show the high prevalence of depression/anxiety and post-traumatic stress disorder of children in immigration detention, with higher behavioural, social and emotional difficulties than the community.^30^ Separating children from families is associated with short- and long-term mental health, social and physical health problems.^31^ Forced separation of children from parents and loved ones is a clear risk factor for adverse mental health. Children's responses to significant stress, such as community violence and persecution experienced by asylum seekers, can be buffered by access to their caretaker and protect against risk. Policies that separate children from families upon entry to the USA can have long-term mental health and developmental consequences, including anxiety, depression, post-traumatic stress disorder, lower IQ, obesity, weakened immune systems, physical growth and morbidity.^32^

Separating children through deportation of parents has profound implications on the children and families growing up without their caretaker, affecting emotional and mental health with changes in sleeping and eating, anxiety, sadness, anger and withdrawal that can last after reunification, along with severed relationships, social belonging and discrimination.^33^ Moreover, expelling unaccompanied children to Mexico while awaiting adjudication of asylum applications places them at undue risk for trafficking, murder, abuse, kidnapping, sexual assault and death.

### Towards the future

The USA has a moral and ethical duty to set an example of how to successfully protect child rights and respect the mental health effects of immigration policies. Building a humanitarian response that protects both country and migrant interest is possible through commitment and policy change that addresses the mental, physical and legal protection needs of children. Below are examples of ways in which policymakers can protect the basic needs, human rights, safety and security of migrant children.

#### Uphold international humanitarian law

First, asylum seekers and refugee children are entitled to humanitarian protection through international law. They have long been stripped of their legal right to pursue asylum for safe haven and protection from persecution, which is in violation of the international principle of non-refoulement.^34^ Children have faced detention and deportation without an asylum hearing or due process.^35^ Any policies, current or future, that allow the harm of children, such as MPP, Title 42 or family separations, should end. Policies that threaten the basic human rights of children and families who cross the border can quickly be enforced. The four principles of the Convention on the Rights of the Child: (a) non-discrimination, (b) best interests of the child, (c) the rights to survival and development, and (d) the views of the child, should be at the centre of immigration policies for children. As well, all children in immigration custody should be appointed legal representation to allow for due process.

#### Shift focus towards protecting the mental health of children

Second, asylum procedures must shift responsibility from law enforcement towards the protection of children's mental health. As the management of migrants into the USA was designed for single men seeking employment, the system must now adapt and respond to the fact that children and families are now more prevalent.^36^ Currently in many parts of the world, law enforcement is managing the welfare of children seeking asylum, instead of those trained and experienced in child welfare. As CBP is not trained in encountering and managing the specific needs of children and families, child welfare professionals and licensed mental health counsellors should be hired to screen children at the border to implement principles that are of best interest to the child. Child welfare professionals that have expertise in human trafficking can focus on mitigating risk of exploitation, prioritising and addressing the mental health needs of youth at CBP stations and shelters. Moreover, as parents and families are often the most proximal sources of support and stress for children, the mental health and well-being of the parents and families surrounding the child must be protected. Studies show the impact of parental mental health in buffering the toxic stress experienced by children.^37^

#### Do no (further) harm

Third, from reception and admission, children should be engaged using a culturally sensitive, trauma-informed approach that seeks to not re-traumatise the child who has likely experienced poly-victimisation and potentially traumatic events. All staff, including enforcement staff such as CBP, need to be trained in understanding the mental health consequences of isolation, separation, fear, ambiguous loss of loved ones and humiliation, that can accompany potentially traumatic events such as deportation, detention and expulsions. Significant reforms in child safety and care while in CBP custody would include mandatory medical and mental health screens, child and adolescent friendly safe spaces, enhanced independent oversight and clear regulations that define acceptable living conditions not only to ensure medical appropriateness, but mental health as well. Children need a safe and clear mechanism for reporting instances of abuse, and should also have routine, daily access to speaking with family and loved ones.

#### Use community stakeholders to end detention

Fourth, children should not be placed in detention settings. Short- and long-term detention of children are a major threat to the normal and optimal psychosocial development of the child, especially without the natural support of parents or parental figures. There is enough evidence and consensus in theory to support that the longer the detention stay, the more damaging.^38^ Across the world, numerous institutions have called for legislative amendments to end immigration detention, particularly of children.^39,40^ Governments should shift care away from law enforcement and realign resources towards those able to provide shelter in the community, such as religious, social service and non-profit organisations that can coordinate efforts and plans for new arrivals seeking asylum. Enhancing and supporting social services can help children and families prepare and appear for court hearings, and support their mental health and well-being while waiting.

#### Advocate to share the burden of responsibility

Fifth, advocacy in shaping the policies that create mental health problems for migrant children would also emphasise the need for countries to collaborate and share the burden of responsibility to address the root causes of migration through promoting economic development, strengthening climate resilience and creating opportunities for social mobility as discussed in the UN High Commissioner for Refugees *Global Compact on Refugees*.^41^ Countries can work together to end corruption, human and drug trafficking, reinforce the rule of law and create regional solutions to allow people safety from violence in their own countries. This sharing of responsibility can also incorporate an increased capacity to manage a humane flow of migration by expanding asylum and humanitarian protection frameworks.

## Discussion

Forced migration is not expected to slow after the pandemic. Climate change with rising sea levels, floods, fires and other natural disasters, will exacerbate drought, food insecurity and poverty. Armed conflict, threats to territories and resource scarcity will continue to fuel humanitarian crises. Now is the time to engage in collaborative action towards building back a more humane, just immigration process for children seeking safe haven, with a focus on basic human rights that include mental health protection.

Mental health is increasingly relevant to not only our social fabric and shared humanity, but also to the economy, and political legitimacy: how we treat those with mental health issues defines the health of our society. These efforts will require an intentional determination to uphold the human rights and dignity of children and families seeking safety, critical reflection of the politics of power and privilege underlying xenophobic policies, and a re-examination of the asylum system in its entirety to ensure a non-punitive, humanistic and just system that places the child at the centre.

## Data Availability

Not applicable.
